# The Ascorbate-glutathione-α-tocopherol Triad in Abiotic Stress Response

**DOI:** 10.3390/ijms13044458

**Published:** 2012-04-10

**Authors:** András Szarka, Bálint Tomasskovics, Gábor Bánhegyi

**Affiliations:** 1 Laboratory of Biochemistry and Molecular Biology, Department of Applied Biotechnology and Food Science, Budapest University of Technology and Economics, 1111 Szent Gellért tér 4, Budapest, Hungary; E-Mail: tomasskovics@mail.bme.hu; 2 Department of Medical Chemistry, Molecular Biology and Pathobiochemistry Pathobiochemistry, Research Group of Hungarian Academy of Sciences and Semmelweis University, 1444 Budapest, POB 260, Hungary; E-Mail: gabor.banhegyi@eok.sote.hu

**Keywords:** abiotic stress, oxidative stress, α-tocopherol, ascorbate, glutathione

## Abstract

The life of any living organism can be defined as a hurdle due to different kind of stresses. As with all living organisms, plants are exposed to various abiotic stresses, such as drought, salinity, extreme temperatures and chemical toxicity. These primary stresses are often interconnected, and lead to the overproduction of reactive oxygen species (ROS) in plants, which are highly reactive and toxic and cause damage to proteins, lipids, carbohydrates and DNA, which ultimately results in oxidative stress. Stress-induced ROS accumulation is counteracted by enzymatic antioxidant systems and non-enzymatic low molecular weight metabolites, such as ascorbate, glutathione and α-tocopherol. The above mentioned low molecular weight antioxidants are also capable of chelating metal ions, reducing thus their catalytic activity to form ROS and also scavenge them. Hence, in plant cells, this triad of low molecular weight antioxidants (ascorbate, glutathione and α-tocopherol) form an important part of abiotic stress response. In this work we are presenting a review of abiotic stress responses connected to these antioxidants.

## 1. Introduction

Under natural conditions, plants are exposed to a variety of biotic and abiotic stresses, including pathogens, adverse temperature, drought, salt, heavy metals and strong light. Under these stress conditions, reactive oxygen species (ROS) derived from molecular oxygen can accumulate in leaves, resulting in the oxidation of cellular components, including nucleic acids, proteins, chlorophyll, and lipids. To cope with oxidative stress, plants have evolved two general functionally interlocked protective mechanisms, enzymatic and non-enzymatic detoxification, of which the latter involves ascorbate, tocopherol and glutathione [1–8].

The levels of these antioxidants are elevated during the fight against ROS. The elevation of these levels can be accomplished by two ways. The up-regulation of the synthesis of these antioxidants is a general response during abiotic stress. On the other hand, the redox recycling of these antioxidants markedly increases their biological efficacy by decreasing the need for *de novo* synthesis. Hence we would like to give a short overview of both mechanisms. The joint discussion of tocopherol, ascorbate and glutathione is reasonable since the three key antioxidants play an interdependent role in the electron transfer stage of the cell due to their recycling [3,8,9].

## 2. ROS Formation in Abiotic Stress

Under optimal growth conditions, ROS are mainly produced at a low level in organelles such as chloroplasts, mitochondria and peroxisomes. However, during stress, their rate of production is dramatically elevated [1–3,8,10].

The chloroplast is considered to be the major source of ROS in plant cells. The photosynthetic fixation of CO_2_ can regulate the generation of ROS. Limited CO_2_ fixation accompanies decreased ATP and NADPH consumption, resulting in an excess of NADPH, especially under strong light. The reduced utilization of NADPH resulted in a decline in the level of NADP^+^. Since NADP^+^ is a major electron acceptor in photosystem I, depletion of NADP^+^ accelerates the transport of electrons from photosystem I to molecular oxygen resulting in the generation of H_2_O_2_ via O_2_^−^. The elevated level of ROS inhibits the repair of damaged photosystem II and leads to photoinhibition [10–13].

Different abiotic stresses such as low and high temperatures, drought, cadmium toxicity and high salinity strongly limit the photosynthetic fixation of CO_2_ and have all been shown to accelerate photoinhibition. It is not so surprising, since moderately elevated temperatures inhibit light activation of Rubisco via the heat-denaturation of Rubisco activase [14,15]. Furthermore, the carboxylation reaction catalyzed by Rubisco is also suppressed by increases in temperature, through a decrease in the specificity of Rubisco for CO_2_ [16]. Cold is known to slow down the Calvin cycle enzymes more than the energy-transducing reactions, also causing NADP^+^ depletion [17]. Under drought conditions, plants close their stomata to prevent water loss by transpiration. Stomatal closure blocks the entry of CO_2_ into leaves resulting in the suppression of photosynthetic carbon fixation even in daytime (during high light condition) [18]. Similarly, nanomolar Cd^2+^ concentrations reduce stomatal opening under light in *Arabidopsis thaliana*, *Vicia faba* and *Commelina communis* in an ABA-independent manner [19].

In the above-detailed limitations of CO_2_ fixation, the carboxylation reaction of Rubisco is suppressed, but the photorespiratory pathway helps to sustain the photosynthetic fixation of CO_2_ in these cases [2]. Photorespiration results in the production of glycolate in chloroplasts. The oxidation of glycolate by glycolate-oxidase occurs in the peroxisomes and accounts for the majority of H_2_O_2_ production during photorespiration [20].

Mitochondrial ROS production is considerably less than ROS generation in chloroplasts or in peroxisomes, due to the high activity of photosynthesis and photorespiration in sunlight. However, in the dark or in non-green tissues, mitochondria are a major source of ROS [21].

The known sites of ROS production in the mitochondrial electron transfer chain (ETC) are complexes I and III. At these complexes (complexes I and III) the ubisemiquinone intermediate is formed, which is the principal electron donor to oxygen, although other complex I sites are also potential donors [22,23]. Hence the extent of mitochondrial ROS production is determined by the overall redox state (reduction level) of the mitochondrial ubiquinone pool. Production of ROS will increase if the rate of electrons leaving the ETC through the terminal oxidases is slowed down and/or the rate of electron input increases in excess of the ability of the two respiratory pathways to process the electrons, leading to an over-reduced ubiquinone pool.

The superoxide anion (O_2_^−^) formed at complex I and III in turn is reduced by dismutation to H_2_O_2_ [24]. H_2_O_2_ can react with reduced Fe^2+^ and Cu^+^ ions to produce highly toxic hydroxyl radicals (OH·) and, being uncharged, can—similarly to H_2_O_2_—penetrate membranes and leave the mitochondrion [23].

Formation of mitochondrial ROS takes place under normal respiratory conditions but can be enhanced in response to a range of abnormal conditions, including exposure to biotic and abiotic stresses [23]. Increased mitochondrial ROS formation due to ETC perturbations was observed in several cases, including chilling [25–27], salt stress [28,29] high temperature (55 °C) [30], exposure to cadmium [30], and phosphate deficiency [31–33].

Mitochondria also interact with chloroplasts and peroxisomes in the photorespiratory cycle to eliminate (mainly by the alternative oxidase pathway) the excess reducing equivalents produced during photosynthesis under conditions of restricted Calvin cycle, thus preventing an over-reduction of the carriers of photosynthetic electron transport [34].

Peroxisomes are the third major sites of intracellular H_2_O_2_ production in plant cells. In the last decade it has been demonstrated that O_2_^−^ (and nitric oxide) radicals are also produced in peroxisomes [35]. Two main sites of O_2_^−^ generation were recognized in plant peroxisomes: In the peroxisomal matrix by xanthine oxidase and in the peroxisomal membrane by a small electron transport chain [36,37]. Xanthine oxidase catalyses the oxidation of xanthine and hypoxanthine into uric acid, and is a well-known producer of O_2_^−^ [35]. The small electron transport chain of peroxisome is composed of a flavoprotein NADH:ferricyanide reductase of about 32 kDa and a cytochrome b [38]. Three integral peroxisomal membrane polypeptides (PMPs) of pea leaf peroxisomes, with molecular masses of 18, 29, and 32 kDa, have been characterized and demonstrated to be responsible for O_2_^−^ generation. The main producer of O_2_^−^ radicals in the peroxisomal membrane was the 18 kDa PMP, which was proposed to be a cytochrome b [37]. In peroxisomal membranes, treatment of pea plants with xenobiotics (clofibrate) induced the 29 kDa polypeptide (PMP29) and depressed the content of PMP32 (very probably corresponding to the monodehydroascorbate reductase, MDAR), and also induced a proliferation of the peroxisomal population of pea and tobacco leaves [38,39].

The main metabolic process responsible for the generation of H_2_O_2_ in different types of peroxisomes is the above-mentioned photorespiratory glycolate oxidase reaction. Besides photorespiration, additional (but minor) sources of H_2_O_2_ production in peroxisomes are fatty acid β-oxidation, the flavin oxidase pathway and the dismutation of O_2_^−^ [38].

## 3. Ascorbic Acid

Low-molecular-weight antioxidants such as ascorbate, glutathione (GSH) and tocopherols can mitigate the above-detailed harmful effects of elevated ROS production. On one hand they can affect gene expression associated with abiotic stresses, altering acclimation responses. On the other hand these antioxidants function as redox buffers that interact with ROS and act as a metabolic interface that modulates the appropriate induction of acclimation responses or programmed cell death [40–42].

In plants, ascorbate is the most abundant antioxidant and also serves as an electron donor to many important reactions [20,43,44]. It generally reaches a concentration of over 20 mM in chloroplasts and occurs in all cell compartments including the cell wall. It is the best known molecule for detoxifying H_2_O_2_, especially as a substrate of ascorbate peroxidase (APX), an essential enzyme of the ascorbate-glutathione cycle, present in most compartments of the plant cell [45]. Control of ascorbate steady-state levels in plants potentially involves regulation of biosynthesis, catabolism, recycling and transport of this compound.

**Figure 1:**
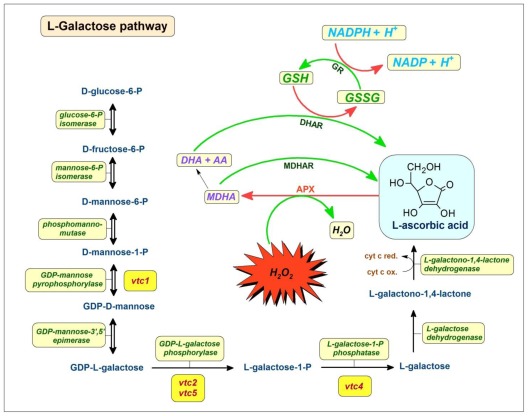
The l-galactose pathway of ascorbate biosynthesis. The l-galactose pathway is the main pathway of ascorbate biosynthesis in plant cells. The phosphorylase reaction is the first committed step in the l-galactose pathway and thus VTC2 and VTC5 are good potential targets for the regulation of ascorbate synthesis. VTC2 (and VTC5) expression have a plateau at the beginning of the light cycle, consequently l-galactose phosphorylase activity rapidly increase on transfer to strong light conditions. Ascorbate supplementation decreased VTC2 expression in Arabidopsis plants, suggesting feedback inhibition at the transcriptional level. Hence GDP-l-galactose phosphorylase may have a major role in controlling ascorbate biosynthesis. Furthermore, the depletion of ascorbate in darkness linked to decreased transcript levels of GDP-d-mannose pyrophosphorylase, l-galactose-1-phosphate phosphatase and l-galactono-1,4-lactone dehydrogenase in the dark. The ascorbate-GSH cycle is also schematically depicted. Oxidations are symbolized by red arrows; reductions are symbolized by green arrows.

Several biosynthetic routes to ascorbate have been proposed, the best established being the pathway through l-galactose [46]. The remaining unknown enzyme in the l-galactose pathway (GDP-l-galactose phosphorylase) has recently been identified [47–49]. There are other suggested routes to ascorbate through galacturonic acid [50,51], l-gulose [52,53]) and myo-inositol [54], but the available evidence from Arabidopsis mutants of the l-galactose pathway genes suggest that the ascorbate derived from these alternate pathways form a relatively small proportion of the total ascorbate pool. The alternative pathways could not compensate for the low levels of ascorbate seen in l-galactose pathway mutants (e.g., vtc-1, vtc-2) [49,55]. More recently, from an analysis of double mutants of the two GDP-l-galactose phosphorylase genes (VTC2 and VTC5) which are lethal to seedlings, it was suggested that the l-galactose pathway is the only significant pathway to ascorbate in Arabidopsis (Figure 1).

Arabidopsis leaves accumulate more ascorbate after acclimatization to high light intensity. VTC2 expression and GDP-l-galactose phosphorylase activity rapidly increase on transfer to a brightly lit environment, but the activity of other enzymes in the GDP-mannose pathway is little affected. VTC2 and VTC5 expression also peak in at the beginning of the light cycle and are controlled by the circadian clock. This observation suggests that GDP-l-galactose phosphorylase may therefore play an important role in controlling ascorbate biosynthesis [47]. It is also supported by the fact that GDP-d-mannose and GDP-l-galactose are not only used for ascorbate formation, but also in the synthesis of cell wall polysaccharides and/or protein glycosylation [44,56], the phosphorylase reaction is the first committed step in the l-galactose pathway and thus VTC2 and VTC5 are good potential targets for the regulation of ascorbate synthesis (Figure 1). Ascorbate, l-galactono-1,4-lactone and l-galactose had no effect on VTC2 activity, indicating no feedback regulation of the enzyme by these metabolites [47]. However, l-ascorbate supplementation decreased VTC2 expression in Arabidopsis plants, suggesting feedback inhibition by ascorbate at the transcriptional level [44]. Taken together, these observations suggest that regulation of VTC2 and VTC5 expression has a major role in controlling ascorbate biosynthesis. This is further supported by the finding that transient overexpression of the kiwi fruit homolog of VTC2 in tobacco leaves led to a threefold increase in ascorbate content, indicating that this enzyme is rate-limiting for ascorbate synthesis [48].

Marked diurnal fluctuations in the leaf ascorbate pool size have been reported with considerable depletion of ascorbate in darkness [57,58] that has been linked to decreased transcript levels of GDP-d-mannose pyrophosphorylase, l-galactose-1-phosphate phosphatase, l-galactono-1,4-lactone dehydrogenase, and the VTC2 gene in the dark [59]. While the light-dependent stimulation of ascorbate biosynthesis appears to require photosynthetic electron transport activity [59], ascorbate synthesis and ascorbate regeneration are influenced by light quality as well as quantity [60]. Ascorbate synthesis and accumulation are particularly sensitive to changes in the light environment, particularly red/far-red ratios, effects that are in line with the direct interactions between the ascorbate pool and the photosynthetic and respiratory electron transport chains [50,52]. Recently, this observation was further supported when a very strong increase in ascorbate contents was also detected in chloroplasts after exposure to strong light by immunohistochemical methods. This data highlights the importance of ascorbate in the antioxidative protection against oxidative stress induced in this cell compartment during conditions of strong light intensity [61].

The beneficial effect of ascorbate during abiotic stress was also shown in mutants of vitamin C biosynthesis. The vtc-1 (synonymous with soz-1) mutant carries a mutation in the gene encoding GDP-mannose pyrophosphorylase involved in ascorbate synthesis, and therefore this plant contains only 30% of wild-type amounts of ascorbate [58,62]. In a study, short-term supplementary UV-B exposure was used to irritate vtc-1 and wild type (WT) Arabidopsis plants. The vtc-1 mutant suffered more damage from UV-B stress than the WT. vtc-1 mutants showed higher levels of lipid peroxidation and H_2_O_2_ production than WT plants. Under the UV-B treatment the content of ascorbate and the ratio of reduced-to-total ascorbate declined more dramatically in the vtc-1 mutants than WT plants, which indicates that the regeneration of reduced ascorbate is also impaired in the vtc-1 mutant. Accordingly, reduced activity of the enzymes responsible for the regeneration of ascorbate and glutathione (including monodehydroascorbate reductase, dehydroascorbate reductase, and glutathione reductase) was reported in vtc-1 mutant [63]. Similarly, the CO_2_ assimilation rate and photosystem II function in the Arabidopsis vtc-1 mutant showed increased sensitivity to salt stress. Moreover, the activity of the ascorbate-glutathione cycle seems to be impaired in the vtc-1 mutant under salt stress. These conditions resulted in more pronounced oxidative stress in the vtc-1 mutant under salt stress than in the WT [64].

Ascorbate, while scavenging ROS and also functioning as an electron donor in various reactions, is oxidized to monodehydroascorbate and then to dehydroascorbate (DHA). DHA is very unstable and only ascorbate possesses antioxidant and free radical scavenger properties. DHA must be reduced back to ascorbate, otherwise under physiological conditions it is lost within minutes. Oxidized ascorbate can be recycled at the expense of glutathione or NADPH by the enzymes of the ascorbate-glutathione cycle: ascorbate peroxidase (APX), monodehydroascorbate reductase (MDHAR), glutathione-dependent dehydroascorbate reductase (DHAR), and glutathione reductase (GR). The elements of the cycle were first described in chloroplast and are often called as Foyer-Halliwell-Asada cycle [65]. The cycle eliminates H_2_O_2_ by the cyclic transfer of electrons without consuming ascorbate or GSH (Figure 1) [66]. Components of this pathway have been shown to be present in animals and in the plant cell cytosol, mitochondria, and peroxisomes as well as the chloroplast [43]. The involvement of complex II, *i.e*., succinate dehydrogenase, in the recycling of ascorbate, has been also demonstrated. DHA, the oxidized transport form of ascorbate, enters mitochondria via a glucose transporter [67] and is subsequently reduced at complex II [68]. Therefore, plant mitochondria sustain not only ascorbate biosynthesis, but also ascorbate regeneration from DHA via multiple mechanisms.

The recycling of ascorbate—similarly to biosynthesis—can provide the appropriate level of ascorbate for the stressed cells. As a proof, the increase in DHAR expression increased foliar and kernel ascorbic acid levels 2- to 4-fold and a significantly increased ascorbate redox state were observed in tobacco and maize [69]. The double CaMV35S promoter fused to the Myc-dhar gene was introduced into *Arabidopsis thaliana* and tested for responses to oxidative stress. In homozygous T(4) transgenic seedlings, DHAR over-expression was increased up to 1.5 to 5.4 fold, which enhanced foliar ascorbic acid levels 2- to 4.25-fold and the ratio of ascorbate/DHA about 3- to 16-fold, relative to wild type. In addition, the level of glutathione, the reductant used by DHAR, also increased as did its redox state. When whole plants were treated with high light and high temperature stress or *in vitro* leaf discs were subjected to 10 μM paraquat, transgenic plants showed a larger ascorbate pool size, lower membrane damage, and a higher level of chlorophyll compared with controls [70]. Similarly, transgenic plants over-expressing MDHAR and/or DHAR showed enhanced tolerance to ozone, drought, temperature and methyl viologen-mediated stresses [71–73]. These results suggested that increasing the plant ascorbate content through enhanced ascorbate recycling could limit the deleterious effects of environmental oxidative stress. Finally, regulation of MDHAR and DHAR involved in ascorbate recycling was investigated in acerola. Under dark conditions, there was a sharp and significant decline in the total and reduced ascorbate contents, accompanied by a decrease in the level of transcripts and enzyme activities of the two genes in acerola leaves. MDHAR and DHAR transcripts and enzyme activities were significantly up-regulated in the leaves of acerola under cold and salt stress conditions, indicating that expression of both genes are transcriptionally regulated under these stresses [74].

## 4. Glutathione

As detailed in the previous chapter, H_2_O_2_ produced by the dismutation of O_2_^−^ in chloroplasts is removed via an ascorbate peroxidase-catalyzed pathway that produces DHA. Although the reduction of dehydroascorbate by glutathione (GSH) is a well-documented chemical reaction, the enzymatic link between ascorbate and glutathione pools is provided by DHA reductase and the enzyme was described several decades ago in plants [75]. DHA reductase was purified and characterized from spinach [76,77] with apparent *K*_M_ values for GSH and DHA of 2.5 and 0.07 mM, respectively, and the reductase was strictly dependent on GSH.

A characteristic feature of GSH is its high concentration in relation to other cellular thiols. In general, GSH accumulates to millimolar concentrations. A second key characteristic of the cellular glutathione pool is its high reduction state. In the absence of stress, tissues such as leaves typically maintain measurable GSH:GSSG ratios of at least 20:1 [78,79]. It is one reason why glutathione is a good candidate to behave as a transmitter of intracellular ROS signals. Furthermore, Foyer and Noctor suggest GSH to the role of redox sensor rather than the ascorbate/DHA redox couple since the redox status of the GSH pool is influenced more intensively by the elevated ROS formation. Moreover, the bulk of the GSSG is localized in the cytosol, but the considerable amount of DHA is probably localized in the apoplast, hence plant cells can maintain low cellular GSH/GSSG and relatively high ascorbate/DHA ratios at the same time [43]. Modulation of plant development in response to stress plays a major role in adaptation of plants to their environment. Mutants defective in thioredoxin reduction and GSH biosynthesis (ntra ntrb cad-2) showed both altered auxin transport and metabolism, which resulted in the loss of apical dominance, vasculature defects, and reduced secondary root production [80].

**Figure 2:**
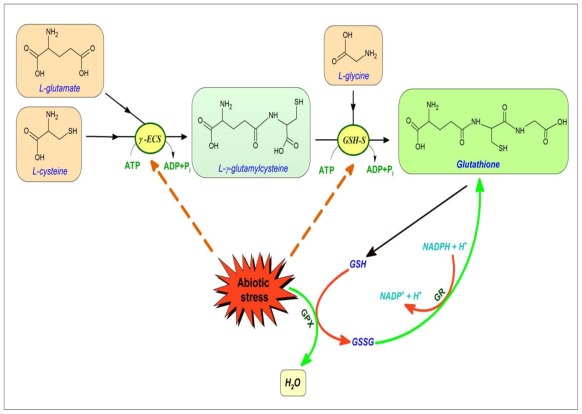
GSH biosynthesis in plants. GSH is synthesized from its constituent amino acids by two ATP-dependent steps. γ-glutamylcysteine ligase (γ-ECS, GSH1) catalyzes the formation of the peptide γ-glutamylcysteine in the second reaction, glutathione synthetase (GSH-S, GSH2) ligates a glycine residue with γ-glutamylcysteine to form GSH. The first step of the biosynthesis has been shown to be a major control point under conditions of increased demand for GSH. The structure of γ-ECS contains two intramolecular disulfide bridges (CC1, CC2) with strong impact on γ-ECS activity *in vitro*. Cysteines of CC2 are involved in the monomer-dimer transition, which is probably an important factor in the up-regulation of GSH synthesis in oxidative stress. Plant γ-ECS shows feedback inhibition by GSH. Cadmium or copper treatments induce the transcription of γ-ECS, GSH-S and GR in Arabidopsis. γ-ECS and GSH-S also respond to jasmonic acid, light, drought and certain pathogens. However, neither externally applied H_2_O_2_ nor intracellularly generated H_2_O_2_ has increased the abundance of γ-ECS or GSH-S transcripts in Arabidopsis. Oxidations are symbolized by red arrows: reductions are symbolized by green arrows.

GSH is synthesized from its constituent amino acids by two ATP-dependent steps. γ-glutamylcysteine ligase (γ-ECS, GSH1) catalyzes the formation of a peptide bond between the γ-carboxyl group of glutamate and the amino group of cysteine, to yield γ-glutamylcysteine. In the second reaction, glutathione synthetase (GSH-S, GSH2) ligates a glycine residue with γ-glutamylcysteine to form GSH (Figure 2). Each of the synthetic enzymes is encoded by a single gene, and Arabidopsis knockout lines for either have lethal phenotypes [79]. The activity of γ-ECS is strongly associated with chloroplasts [81]. Localization studies in Arabidopsis have demonstrated that the enzyme is restricted to plastids in this species [82]. Arabidopsis GSH-S is found in both chloroplasts and cytosol. The first step of glutathione synthesis is plastidic while the second step is predominantly located in the cytosol [77]. Inner chloroplast envelope transporters have recently been described that likely act to link plastidic γ-ECS and cytosolic GSH-S via γ-EC export across the chloroplast envelope [83]. In the light of this observation, it is intriguing that mitochondria always showed highest amounts of glutathione, whereas plastids contained the lowest amounts of glutathione [84]. Similar results were obtained in previous studies which have additionally revealed that mitochondria maintain high and stable levels of glutathione even in situations of temporary and permanent glutathione deficiency [85,86]. Peroxisomal glutathione concentration is similar to the cytosolic concentration [83], and it has been estimated in leaf mesophyll cells to be around 3–4 mM [87]. Presumably, similar to the mitochondrial, this pool results from import across the peroxisomal membrane. Both transporters responsible for this activity remain to be characterized.

The first step of the biosynthesis has been shown to be a major control point under conditions of increased demand for GSH. The recent elucidation of γ-ECS structure from *Brassica juncea* has revealed the presence of two intramolecular disulfide bridges (CC1, CC2), which both strongly impact on γ-ECS activity *in vitro*. Cysteines of CC2 are involved in the monomer-dimer transition. γ-ECS from tobacco forms a homodimer under oxidizing conditions, and is activated more than threefold [88]. This is probably an important factor in the well-known up-regulation of glutathione synthesis in response to oxidative stress. It should also be noted that γ-ECS in plants (similar to the animal counterpart) shows feedback inhibition by GSH [79].

Heavy metals induce the synthesis of organic ligands that could form metal complexes with reduced biological activity. Among these compounds, phytochelatins (PCs) are known to bind cadmium and other toxic elements by means of sulfhydryl residues that are then transported into the vacuole [89]. Phytochelatins are synthesized from GSH and homologous biothiols by the enzyme phytochelatin synthase (PCS) [90–93]. When plants are exposed to heavy metals, PCS condenses the γ-glutamyl-cysteine moiety of a GSH molecule with the glutamic acid residue of a second GSH, releasing glycine and increasing the length of the PC molecule [94,95]. Arabidopsis plants treated with cadmium or copper responded by increasing transcription of the genes for glutathione synthesis and reduction, γ-ECS and GSH-S, as well as GR. The response was specific for those metals whose toxicity is thought to be mitigated through phytochelatins, and other toxic and nontoxic metals did not alter mRNA levels [96]. Feeding experiments suggested that neither oxidative stress resulting from exposure to H_2_O_2_, nor oxidized or reduced GSH levels were responsible for activating transcription of these genes, despite the well-described increases in GSH in these conditions. Jasmonic acid also activated the same suite of genes, which suggests that it might be involved in the signal transduction pathway for copper and cadmium. Jasmonic acid treatment increased mRNA levels and the capacity for glutathione synthesis but did not alter the glutathione content in unstressed plants. γ-ECS and GSH-S also respond to light and some stress conditions such as drought and certain pathogens [96]. In accordance with the observation that enhanced cysteine supply favors glutathione accumulation, increases in GSH synthesis are associated with up-regulation of the cysteine synthesis pathway. For example, GSH accumulation triggered by oxidative stress causes accumulation of transcripts encoding adenosine 5′-phosphosulfate reductase and serine acetyltransferase [79,97].

Over-expression of *Escherichia coli* GSH-S in poplar produced little effect on glutathione contents in optimal conditions [98,99], introduction of the *E. coli* γ-ECS caused a 2- to 4- fold increase in leaf glutathione, and this was observed whether the bacterial γ-ECS was targeted to the cytosol or the chloroplast [66,99,100]. Expression of the same γ-ECS in the tobacco chloroplast also produced substantial increases in leaf glutathione in the experiments of Creissen *et al.* [101]. Transgenic tobacco plants showed chlorosis and necrosis in response to high light intensity, which paradoxically resulted from increased oxidative stress. The transgenic tobacco exhibited a shift in the redox state of the GSH and γ-EC pool to a more oxidized state, which was accompanied by enhanced H_2_O_2_ levels that either occurred from increased production or defective ROS scavenging [101]. The authors concluded that the low redox state disturbed redox-sensing processes in the chloroplasts. Interestingly, this phenotype has not been observed in young poplar over-expressing the same bacterial γ-ECS gene targeted to chloroplasts [66], even though the increase in GSH was within the same range. The authors concluded that these differences could be caused by different growth habits of these species [101]. Recently Liedschulte *et al.* reported the expression of the bifunctional γ-glutamylcysteine ligase-glutathione synthetase enzyme from *Streptococcus thermophilus* (StGCL-GS) in tobacco, which was shown to be neither redox-regulated nor sensitive to feedback inhibition by GSH [102]. Transgenic tobacco plants expressing StGCL-GS under the control of a constitutive promoter reveal an extreme accumulation of GSH in their leaves (up to 12 μmol GSH/gFW, depending on the developmental stage), which is more than 20- to 30-fold above the levels observed in wild-type plants and which can be even further increased by additional sulfate fertilization. Surprisingly, this dramatically increased GSH production has no impact on plant growth while enhancing plant tolerance to abiotic stress. To date, no marked deleterious effects have been reported [79]. An important difference could be in the work of Creissen *et al.* [101] and Liedschulte *et al.* [102] that, unlike the *E. coli* γ-ECS, the streptococcus protein has both GSH-S and γ-ECS activities. Several studies have shown the benefits of elevating GSH through over-expression of γ-ECS. These include enhanced resistance to heavy metals and certain herbicides [103–105].

The chemical reaction of GSH with H_2_O_2_ is slow, but three distinct types of peroxidases appear as the principal candidates to link peroxide reduction to GSH oxidation. These are ascorbate peroxidase (APX), certain types of peroxiredoxin (PRX) and glutathione *S*-transferases (GSTs). Among these, only GSTs appear to act as direct glutathione peroxidases (GPXs): all other enzymes requiring at least one additional protein to link peroxide reduction to GSH oxidation. The evidence from gene expression makes it clear that certain APX, GPX and GST genes are induced in response to oxidative stress [106–109]. GSSG produced by GSH oxidation is reduced by GR. Despite the long-standing association of GR and GSH with resistance to various stresses [110,111], over-expression of GR in itself has not been reported to lead to marked increases in stress resistance in several plant species [97,112–116]. However, increases in the reduction state of the ascorbate pool in plants over-expressing GR are consistent with efficient coupling of the reactions of the ascorbate-glutathione pathway [117].

## 5. Vitamin E

Plant tissues vary enormously in their total tocopherol content and tocopherol composition, with total concentrations ranging from extremely low levels in the potato tuber (<1 μg/g dry weight) to very high levels in leaves and seeds (>1 mg/g dry weight) [118].

All vitamin E compounds (tocopherols and tocotrienols) are formed by a chromanol head group and a prenyl side chain. All tocopherols and tocotrienols are amphipatic molecules in which the hydrophobic prenyl tail associates with membrane lipids and the polar chromanol head groups are exposed to the membrane surface. Tocopherols differ from tocotrienols only in the degree of saturation of their hydrophobic tail, and the α, β-, γ-, δ-forms of tocopherols and tocotrienols vary only in the number and position of methyl substituents attached to the chromanol ring [119].

α-Tocopherol is synthesized in the envelope of plastids [120,121], and is stored in plastoglobuli of the chloroplast stroma, [122,123], and in thylakoid membranes [124,125]. Most of the α-tocopherol synthesized is partitioned between the chloroplastic envelope and the thylakoids and is stored in plastoglobuli only in some cases. In spinach chloroplasts, one-third of the total α-tocopherol is located in envelope membranes, and the remaining two-thirds in thylakoids [126].

**Figure 3:**
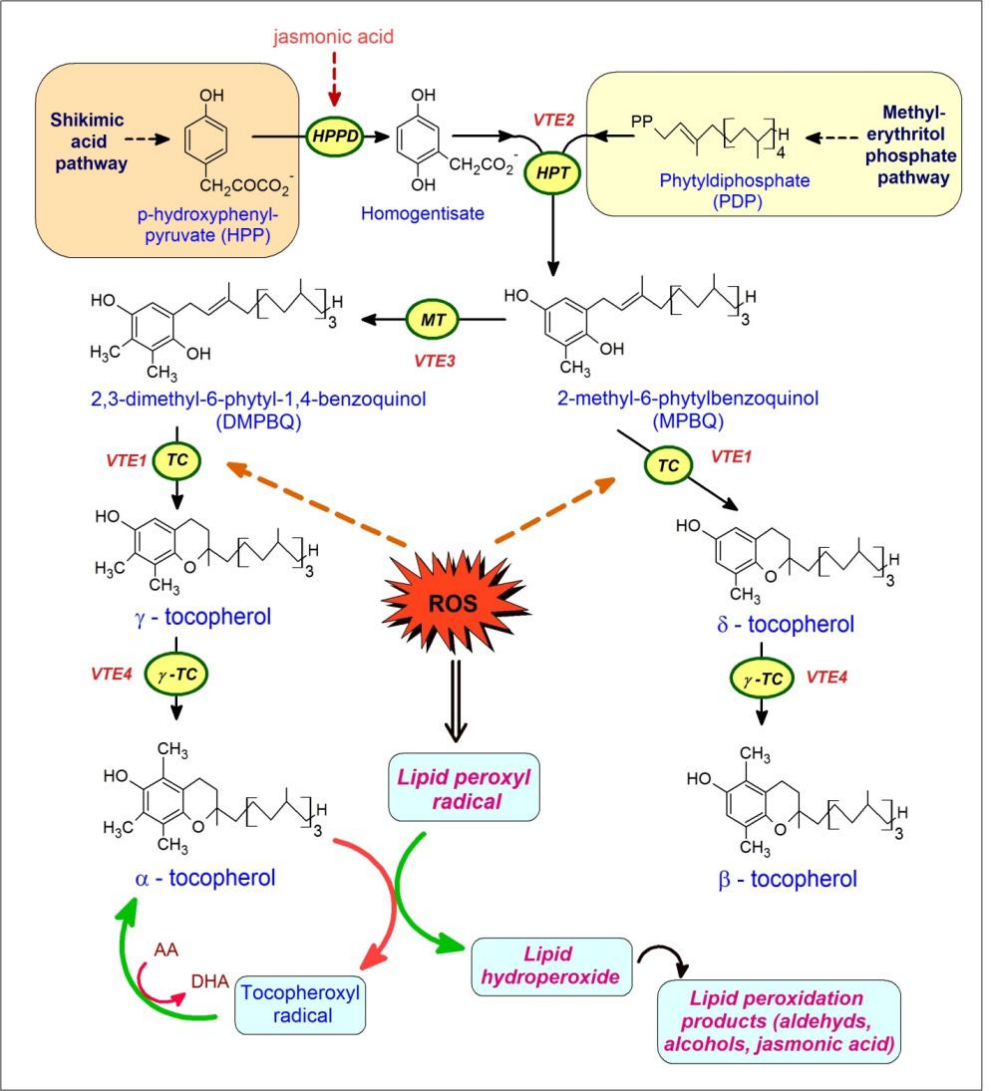
Tocopherol/tocotrienol biosynthesis in plants. The hydroquinone ring of tocopherol is derived from the shikimate pathway of aromatic amino acid synthesis. Homogentisate, the precursor for the synthesis of tocopherol, tocotrienol, and plastoquinone, is synthesized by *p*-hydroxyphenylpyruvate dioxygenase (HPPD). After attachment of the hydrophobic side chain by homogentisate phytyltransferase (HPT1/VTE2) and methylation, 2,3-dimethyl-5-phytyl-1, 4-hydroquinol (DMPQ) is formed that is converted to γ-tocopherol by tocopherol cyclase (VTE1). Final methylation by γ-tocopherol methyltransferase (γ-TMT, VTE4) results in the production of α-tocopherol. Tocopherol synthesis is regulated in plants via environmental stress, and stress sensitive hormones such as jasmonic acid, salicylic acid and abscisic acid (ABA). Two major points of regulation were identified: 1. HPPD is regulated by jasmonic acid and ABA, 2. VTE1 is strongly induced during oxidative stress. Oxidations are symbolized by red arrows: reductions are symbolized by green arrows.

The hydroquinone ring of tocopherol is derived from the shikimate pathway of aromatic amino acid synthesis. Homogentisate, the precursor for the synthesis of tocopherol, tocotrienol, and plastoquinone, is synthesized by *p*-hydroxyphenylpyruvate dioxygenase (HPPD) [127] (Figure 3). After attachment of the hydrophobic side chain by homogentisate phytyltransferase (HPT1/VTE2) [128,129] and methylation (VTE3) [130,131], 2,3-dimethyl-5-phytyl-1,4-hydroquinol (DMPQ) is formed that is converted to γ-tocopherol by tocopherol cyclase (VTE1) (Figure 3) [132,133]. An increase in the activity of HPPD or HPT1 in transgenic plants resulted in an elevated tocopherol content in seeds and leaves of Arabidopsis [134,135]. It was concluded that flux into tocopherol is predominantly controlled by HPPD and HPT1, but the biosynthetic steps further downstream in the pathway, e.g., VTE1, are not limiting [135]. However, the observations of Kanwischer *et al*. suggest that VTE1 is strongly induced during oxidative stress and that it is a major factor limiting tocopherol synthesis in leaves [136]. Final methylation by γ-tocopherol methyltransferase (γ-TMT, VTE4) results in the production of α-tocopherol. α-Tocopherol is the predominant form in leaves, whereas γ-tocopherol is most abundant in seeds of Arabidopsis [137].

The antioxidant activity of tocopherols and tocotrienols as free-radical scavengers is associated with the ability to donate its phenolic hydrogen to lipid free radicals, and with specific requirements of the molecule. These are the degree of methylation in the aromatic ring (α > β = γ > δ), the size of the heterocyclic ring, the stereochemistry at position 2, and finally the length of the prenyl chain (optimum between 11 and 13 carbons). It has been shown that the antioxidant activity of tocopherols and tocotrienols has two main roles [118]. First tocopherols and tocotrienols scavenge the lipid peroxy radical before it can abstract hydrogen from the target lipids. The chromanol rings of tocopherols and tocotrienols lose a hydrogen atom, which is given to the lipid peroxy radical, and tocopheroxyl or tocotrienoxyl radical are formed. However, in the absence of recycling of tocopheroxyl and tocotrienoxyl radicals by ascorbic acid and glutathione, the radicals may undergo radical-radical coupling with other lipid peroxy radicals to form adducts, and may convert to form quinones or may undergo self-coupling with other tocopheroxyl and tocotrienoxyl radicals to form dimers and/or trimers [138]. Secondly, tocopherols and tocotrienols also play a key role as antioxidants because they physically quench or chemically scavenge singlet molecular oxygen (^1^O_2_), the excited molecular oxygen with spin paired valence electrons. One molecule of α-tocopherol can deactivate up to 120 ^1^O_2_ molecules by resonance energy transfer [139].

It can be assessed that stress-tolerant plants usually display increase tocopherol levels, but the most sensitive ones show net tocopherol loss under stress, which leads to oxidative damage and cell destruction [118,140]. Several observations support this state, e.g., α-tocopherol increases remarkably by water deficit in spinach and pea leaves [141,142], in wheat and other grasses [143,144], in Mediterranean shrubs such as rosemary and lavender [145,146], and in European beech seedlings [147]. However an interesting observation can be also taken; the changes in α-tocopherol level during plant responses to environmental stress are characterized by two phases. In the first phase, there is an increase in tocopherol synthesis, which is followed by a second phase of net tocopherol loss [140]. Hence, it is not surprising that rice seedlings cultured hydroponically and subjected to water stress in 30% polyethylene glycol showed a loss of α-tocopherol in chloroplasts [148]. These results also indicate that although α-tocopherol may afford a certain degree of protection against UV-B radiation, this protection is limited by the amount of other antioxidants present in membranes and/or by the molecular species of reactive oxygen.

As in the cases of the other two antioxidants, the level of tocopherol is the result of synthesis, recycling and degradation (consumption).

Tocopherol synthesis is regulated in plant responses to environmental stress, and stress sensitive hormones such as jasmonic acid, salicylic acid and abscisic acid (ABA) appear to play a role. It has been shown that the expression of tocopherol biosynthetic genes, particularly those encoding for tyrosine aminotransferase (tat) and HPPD, is regulated by jasmonic acid [149,150]. Similarly, a strong positive correlation between salicylic acid and α-tocopherol has been observed in field-grown *Phillyrea angustifolia* plants exposed to drought stress [151]. Moreover, an abscisic acid-specific motif has been identified in the promoter region of the HPPD gene, which indicates that tocopherol biosynthesis may be stimulated by ABA [140].

The important role of VTE1 in tocopherol biosynthesis, mentioned earlier, has been further supported by transgenic approaches. Transgenic tobacco plants over-expressing VTE1 from Arabidopsis exposed to drought conditions showed decreased lipid peroxidation, electrolyte leakage and H_2_O_2_ content, but had increased chlorophyll compared with the wild type [152]. In addition to its enhanced ability to scavenge ROS and to avoid oxidative damage, VTE1 transgenic plants with higher tocopherol content also exhibited improved membrane integrity, and resulted in more controlled water efflux during drought conditions [152].

The crucial role of vitamin E in the tolerance of Arabidopsis to heavy metals which induce (75 μM Cd^2+^ or 75 μM Cu^+^) oxidative stress was also described. Transcripts encoding enzymes of the vitamin E biosynthetic pathway increased in response to metal exposure. In particular, VTE2 mRNA was enhanced in Cu^+^- and Cd^2+^-treated plants. Accordingly, the vitamin E-deficient (vte-1) mutant exhibited an enhanced sensitivity towards both metals relative to the wild-type control [153].

Besides the rate of biosynthesis, the endogenous α-tocopherol levels are also severely affected by the extent of its degradation and recycling under stress. As stress is more severe and the amounts of ROS in chloroplasts increase, α-tocopherol levels tend to decrease. While quenching of ^1^O_2_ by α-tocopherol (the deactivatation of ^1^O_2_ molecules by resonance energy transfer) does not lead to a significant degradation of this antioxidant, chemical scavenging of ^1^O_2_ by α-tocopherol can lead to a net tocopherol loss, since α-tocopherol quinone and other oxidation products formed cannot be recycled back to α-tocopherol [118].

Irreversible degradation of α-tocopherol may also occur when α-tocopheroxyl radicals, which result from the scavenging of lipid peroxyl radical by α-tocopherol, are not recycled back by ascorbate. This may occur, when ascorbate is limited in chloroplasts, as occurs in vtc-1 mutants of Arabidopsis. Although these mutants display similar [154] or even enhanced [136] tocopherol levels under non-stress conditions, they show α-tocopherol loss under stress caused by a severe deficiency of ascorbate in chloroplasts [155].

## 6. Conclusions—Or Lessons to the Human Being from the Plant Cell

**Figure 4:**
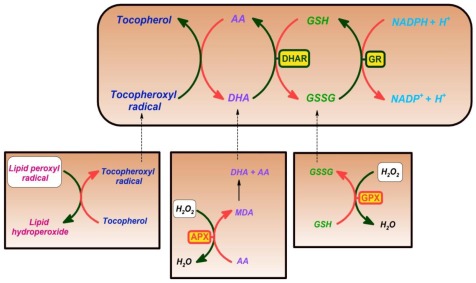
The interdependent ROS scavenging network of the α-tocopherol-ascorbateglutathione triad. Tocopherols scavenge the lipid peroxy radical before it can abstract hydrogen from the target lipids. Tocopherols lose a hydrogen atom, which is given to the lipid peroxy radical, and tocopheroxyl radical is formed. The tocopheroxyl radical is reduced by ascorbate (Asc), while dehydroascorbate (DHA) is formed. Ascorbate is also involved in the detoxifying process of H_2_O_2_, especially as a substrate of ascorbate peroxidase (APX), which generates two molecules of monodehydroascorbate (MDHA). MDHA may be reduced to ascorbate by the catalysis of monodehydroascorbate reductase (MDAR). MDHA may also rapidly oxidize to DHA, which is then reduced to ascorbate by the action of dehydroascorbate reductase (DHAR) with glutathione (GSH) as a substrate, generating glutathione disulfide (GSSG). The alternative way of H_2_O_2_ elimination can be performed by glutathione peroxidase (GPX) at the expense of GSH. Finally, glutathione reductase (GR) reduces GSSG to GSH on the expense of NADPH.

Ascorbate and glutathione are the two major soluble antioxidants in plant cells, and they are linked via the well documented ascorbate-glutathione cycle. The ascorbate-glutathione cycle was implicated in the reduction of the tocopheroxyl radical to tocopherol (Figure 4) [124,140]. *In vitro* experiments showed that the tocopherol-mediated protection against lipid peroxidation is strongly enhanced by the presence of ascorbate and glutathione [155]. Elegant evidence of the interplay between hydrophilic and lipophilic antioxidants has been given by vtc-1 mutant Arabidopsis chloroplasts during drought stress. Although low ascorbate did not cause oxidative stress in optimal growth conditions, it increased malondialdehyde levels in chloroplasts by 60%, and reduced tocopherol by 85% in water-stressed mutants [154]. The parallel degradation of tocopherol and enhanced lipid peroxidation in chloroplasts of water-stressed mutants clearly demonstrates the significance of ascorbate on the antioxidant defense system and is indicative of the interplay between hydrophilic and lipophilic antioxidants. Similar cooperation between hydrophilic and lipophilic antioxidants could also be described in Cu-exposed Arabidopsis leaves. The accumulation of α-tocopherol—the major vitamin E component in leaves— during Cu stress was accompanied by the marked rise in the level of ascorbate [153]. The coordinated elevation of ascorbate may increase the capacity of tocopherol recycling and hence the antioxidant capacity of tocopherols during Cu-induced oxidative stress. The synergistic antioxidant effect of the triad was also supported by the observation that under conditions of intense light, the levels all antioxidants (tocopherol, GSH, and ascorbate) increased several fold in a coordinative manner [136]. Similarly, the coordinated activation of the enzymes of ascorbate-GSH cycle and elevated GSH level could also be observed in ppr-40 mutant Arabidopsis, characterized by low ascorbate level due to mitochondrial complex III deficiency [65].

The absence of one or more of these three antioxidants in plant mutants in the biosynthesis of one or two of the other above-mentioned antioxidants (vtc-1, cad-2, vte-1) leads to an increase in oxidative stress in the plant cell, and as a consequence, the amounts of the remaining antioxidants increase. On the other hand, a high tocopherol content resulted in a reduction of ascorbate and GSH in VTE1 overexpression lines [136].

These observations provide strong evidence that the objective of avoiding oxidative damage can be achieved more easily and efficiently by joint effort.
